# Lactopeptine

**Published:** 1879-02

**Authors:** 


LACTOPEPTINE is now a favorite prescription with many physicians in cases of Indigestion, Dyspepsia and other derangements of digestion, and seems to be also growing in favor with the public, though it is a remedy dependent upon the profession alone for its success. We have given it a trial, and patients speak of its effects most favorably in many cases. It seems worthy of a trial, at least, indicated, as it is in a wide range, of functional derangements.

